# Evaluation of the effect of vitamin D supplementation on spermatogram, seminal and serum levels of oxidative stress indices in asthenospermia infertile men: a study protocol for a triple-blind, randomized controlled trial

**DOI:** 10.1186/s12937-021-00711-7

**Published:** 2021-06-02

**Authors:** Leila Maghsoumi-Norouzabad, Ahmad Zare Javid, Anahita Mansoori, Mohammadreza Dadfar, Amirarsalan Serajian

**Affiliations:** 1 Department of Nutrition, School of Allied Medical Sciences, Ahvaz Jundishapur University of Medical Sciences, Ahvaz, Iran; 2 Nutrition and Metabolic Diseases Research Center, Ahvaz Jundishapur University of Medical Sciences, Ahvaz, Iran; 3 Department of Urology, Imam Khomeini Hospital, School of Medicine, Ahvaz Jundishapur University of Medical Sciences, Ahvaz, Iran; 4 Health education group, Jahad Daneshgahi, Ahvaz, Khuzestan Iran

**Keywords:** Vitamin D, Oxidative stress, Infertile men, Idiopathic asthenospermia, Semen quality, Sex hormones

## Abstract

**Background:**

It is suggested that vitamin D may have a beneficial role in male reproduction. The male reproductive system is a target tissue for vitamin D. This study will aim to evaluate the effects of vitamin D supplementation on sperm parameters, seminal and serum levels of oxidative stress and serum endocrine factors in asthenospermia infertile men.

**Methods/design:**

This randomized, triple-blind, placebo-controlled clinical trial will be conducted on 86 infertile men with idiopathic asthenozoospermia (the mobility of sperm < 40% and rapid progressive sperm motility < 32%), with serum levels of vitamin D less than 30 ng / ml in the “Infertility Clinic of Ahvaz Jahad daneshgahi”, Iran.

**Main outcomes measure (s):**

Demographic data, dietary intake, physical activity, sun exposure, anthropometric indices, serum and seminal levels of MDA (Malondialdehyde), 8-hydroxy-2- Dioxy Guanosine (8-OHDG), Total Antioxidant Capacity (TAC) and calcium, sperm DNA fragmentation index (DFI), serum 25-OHD, luteinizing hormone (LH), follicle-stimulating hormone (FSH), total testosterone (T), estradiol (E2), sex hormone-binding globulin (SHBG), free androgen index (FAI = T/SHBG. 100), T/LH and T/E2 ratios, prolactin (PRO), parathyroid hormone (PTH), osteocalcin (OCN), phosphorus and sperm parameters.

**Discussion:**

The deficiency of vitamin D as an antioxidant is common all over the world. Numerous observational studies have shown a positive association between vitamin D levels and semen quality. However, few clinical studies have been conducted in this area. So considering with the high prevalence of this antioxidant deficiency specifically in infertile men, it seems that the supplementation of vitamin D in infertile men with insufficient levels or deficiency may improve the status of oxidative stress and thereby may affect sperm parameters and endocrine factors involved in male fertility.

**Trial registration:**

Iran Clinical Trials Registry, ID: IRCT20151128025274N4, registered on 28 March 2018.

**Supplementary Information:**

The online version contains supplementary material available at 10.1186/s12937-021-00711-7.

## Introduction

Infertility is defined as the inability to have a child after at least 1 y of marriage without using any preventive method (WHO). About 15% of couples across the world suffer from infertility [1]. The prevalence of infertility is 24.9% among Iranian couples, which is greater than the global average [2]. It is indicated that about half of the causes of infertility is attributed to the male partner [1]. Nineteen percent of infertile cases have asthenospermia, the impaired sperm motility. Persistent poor motility predicts failure in fertilization [1].

The oxidative stress (OS) caused by increased production of reactive oxygen species (ROS) in semen have been suggested as an important causative factor in the etiology of asthenospermia [3]. Spermatozoa were the first cells in which the production of ROS was reported. Macleod (1943) reported that the incubation of spermatozoa at high oxygen pressures slowed down the sperm motility due to increased production of H_2_O_2_ [4]. It should be noted that ROS, at low physiological concentrations, are required for maturation, capacitation, hyper activation of sperm, the acrosome reaction, and fertilization [5]. However, the excessive production of ROS may lead to serious damage to the cell function [6]. Immature and abnormal spermatozoa, along with positive peroxidase leukocytes (neutrophils and macrophages) are considered as two major endogenous sources of continuous ROS production [7]. Researches showed that ROS and their metabolites can attack Deoxyribonucleic acid (DNA), lipids, and proteins and may lead to alter enzymatic systems; produce irreparable alterations; cause cell death and ultimately impair the semen parameters associated with male infertility. It is known that spermatozoa are susceptible to oxidative damage as their plasma membranes are rich in polyunsaturated fatty acids and have low concentrations of scavenging enzymes [8]. Studies have shown that the concentration of malondialdehyde (MDA), which is an indicator of lipid peroxidation, is twice as high in infertile men with asthenospermic and oligoasthenospermic infertility compared with men with normospermic [9].

Many researchers suggested that measuring 8-hydroxydeoxyguanosine (8-OHdG), a product of DNA oxidation, in sperm and seminal fluid can be a proper and direct indicator of oxidative damage of sperm DNA [10]. DNA damage by active oxygen species can lead to the transmission of defective paternal DNA to the fetus. One of the common methods to measure the damage on sperm DNA is the SCD test in which the amount of denatured DNA is determined. The amount of denatured DNA is evaluated using DNA fragmentation index (DFI). Studies have shown that the DFI levels above 27% are associated with not only the increased fertility failure but also the failure of assisted reproductive techniques [11, 12]. In addition, the inflammation caused by free radicals can reduce the production of testosterone (T) and Luteinizing hormone (LH) and thereby affect fertility [13–15]. There are series of enzymatic antioxidants and low molecular weight antioxidant compounds in seminal plasma and sperm, which can protect the sperm against the ROS damage [10, 16, 17]. Studies found that the antioxidant capacity of seminal fluid in infertile men is lower than fertile men [18, 19].

There are several treatment approaches for infertility including surgery, prescription of medications for the production and mobility of sperm and the artificial fertility, which the most are expensive. Moreover, there are some alternative cost-effective methods such as using food supplements with antioxidant properties, reported to increase the sperm count and motility [20–22].

Vitamin D is considered as an important micronutrient with many biological effects. Vitamin D deficiency is the epidemic concern of modern age and the most common nutritional deficiency worldwide [23, 24]. The expression of vitamin D receptors (CYP2R1, CYP27B1 and CYP24A1) and enzymes involved in the metabolism and activation of vitamin D in testicles specifically in leydig cells, in epididymis, seminal vesicles, prostate and spinal cord and the head of sperm indicate the importance of the role of vitamin D in fertility and reproduction in men [22]. According to evidence, vitamin D levels are greater in fertile men than in infertile men. Also, there is an association between vitamin D deficiency and low sperm quality in infertile men according to several studies [25–27]. In addition, animal studies showed that vitamin D deficiency is linked to an increase in sperm DNA fragmentation [28]. Deng XL et al. showed that the 3 months supplementation with Vitamin D and calcium in oligo-asthenozoospermia infertile men significantly increased sperm progressive motility and rate of pregnancy in the intervention group [29]. In contrast Blomberg Jensen et al. [30] and Amini et al. [31] found no significant differences in sperm parameters between two groups post intervention in the RCTs.

Furthermore, the impact of vitamin D deficiency on serum levels of testicular hormones have been assessed by several animal, observational and interventional studies with controversial results regarding with clear relationship between vitamin D status and testicular hormone production [32–49].

### Objectives and hypotheses of the study

Based on the evidence indicating that the expression of vitamin D receptors and enzymes are involved in the metabolism of vitamin D in genital and male sperm, it is hypothesized that vitamin D may have potential role in spermatogenesis, sperm maturation, endocrine functions and thereby in improvement of male fertility. Moreover, regarding with the role of OS in the etiology of poor semen quality and male infertility, and also considering the antioxidant role of vitamin D and high prevalence of vitamin D deficiency in Iran, we propose that vitamin D supplementation may improve the OS, sperm parameters such as sperm motility and endocrine factors involved in male fertility. To the best of our knowledge, in the most studies conducted on the effects of antioxidants on infertility, some antioxidants such as vitamin E, selenium and vitamin C were used as supplements. There are only few clinical trials with controversial results evaluated the effects of vitamin D supplementation on male infertility. Furthermore, most of these studies used the adjunctive supplementation of Vitamin D and calcium. Also, there is no clinical study has examined the effect of vitamin D supplementation on OS markers in men with asthenospermia and insufficient vitamin D levels or vitamin D deficiency. Therefore, it is suggested that further clinical studies are needed in this field. So, the main objective of this study is to evaluate the effects of vitamin D supplementation on sperm parameters, seminal and serum levels of OS markers and also serum endocrine factors in asthenospermia infertile men.

## Methods/design

### Study subjects

This trial will be conducted on 86 infertile men with idiopathic asthenozoospermia (the mobility of sperm < 40% and rapid progressive sperm motility < 32% [50], and with serum vitamin D levels < 30 ng / ml, who report inability to have a child after at least 1 y of marriage without using any preventive methods and with normal fertile female partner in the Infertility Clinic of Ahvaz Jahad daneshgahi, Iran. The further inclusion criteria are listed in Table 1.

**Table 1:** Inclusion and exclusion criteria

Inclusion	
•Men aged 20 to 50 years	
•Idiopathic asthenozoospermia (the mobility of sperm < 40% and rapid progressive sperm motility < 32%	
•Serum vitamin D levels < 30 ng / ml	
•Access to the infertility clinic of Ahvaz Jahad daneshgahi, Iran	
•Inability to have a child after at least 1 y of marriage without using any preventive methods	
•Normal fertile female partner	
•No history (≤ 12 weeks) of medical therapy	
•Not having a history of epididymo-orchitis, prostatitis, genital trauma, testicular torsion, inguinal or genital surgery, urinary tract infection, or previous hormonal therapy, another genital disease (cryptorchidism, current genital inflammation or varicocele), a recent history of sexually transmitted infection, hepatobiliary disease and renal insufficiency, severe general or central nervous system disease and endocrinopathy, using cytotoxic medications, immunosuppressant, anticonvulsants, androgens, antiandrogens	
•No smoking, drug or alcohol abuse, and	
•No occupational and environmental exposure to possible reproductive toxins	
•Body mass index of < 30 kg/m^2^	
Exclusion	
• Have any acute illness	
• Use less than 90% of the prescript supplement	
• Would like to leave the study personally	
• Participate in another study	
• Immigrate	
• Not available for following-up	

### Design

**Fig. 1:**
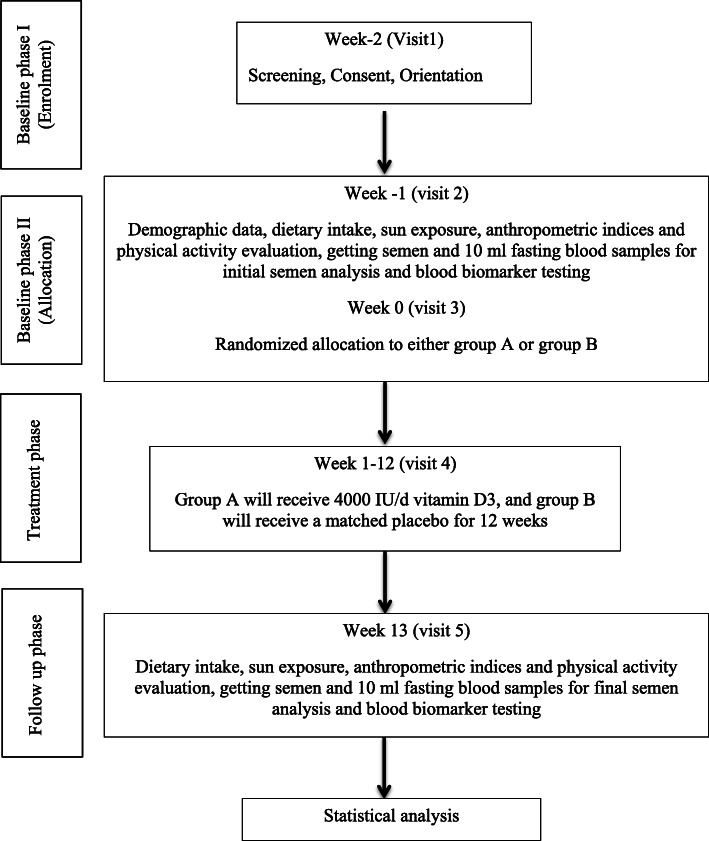
Flow diagram of the study

This study is a randomized, triple-blind, placebo-controlled clinical trial. There will be four study phases consisting of baseline phase I “enrolment”, baseline phase II “randomization and allocation”, treatment and follow up over 12 weeks (Fig. 1). Participants will be randomly allocated into two groups of either receiving 4000 IU/d [51, 52] vitamin D3, or a matched placebo. Participants, the researchers, and statistical counselor will be blinded in terms of the type of intervention throughout the trial. All visits will be held in the “Infertility Clinic of Ahvaz Jahad daneshgahi”, Iran during the day.

### Primary aim

The primary aim of the present study is to improve serum levels of 25OHD3 through vitamin D3 supplementation in order to evaluate its effects on fertility in infertile men. The compliance of subjects will be monitored by counting the remaining supplements. The subjects who may not use greater than 90% of the supplements will be excluded. We expect that the drop-out rates will be high in this study. Therefore, we would consider a 25% attrition rate for each group of study.

### Secondary aims

The secondary aims of this study are as follow:

1. To determine the effects of vitamin D supplementation on the status of oxidative stress in asthenospermia infertile men with vitamin D deficiency
2. To determine the effects of vitamin D supplementation on endocrine factors involved in fertility in asthenospermia infertile men with vitamin D deficiency

### Practical aims

Vitamin D supplementation will be recommended for asthenospermia infertile men, if its beneficial effects would be confirmed in this study. Also, in order to increase dietary intakes of vitamin D in infertile men, there will be necessary recommendations provided for these patients.

### Primary outcomes

The primary outcomes are as follow:

1. Changes in the mean serum levels of 25-OHD between and within two groups pre and post intervention in asthenospermia infertile men
2. Changes in the mean ejaculation volume, total sperm count, percentage of motile sperm, number of sperm with normal morphology, number of live sperm in semen between and within two groups pre and post intervention in asthenospermia infertile men.

### Secondary outcomes

Secondary outcomes are:

1. Changes in the mean serum and seminal levels of MDA (Malondialdehyde), 8-hydroxy-2- Dioxy Guanosine (8-OHDG), total antioxidant capacity (TAC), and sperm DNA fragmentation index (DFI) between and within two groups pre and post intervention in asthenospermia infertile men
2. Changes in the mean serum levels of luteinizing hormone (LH), follicle-stimulating hormone (FSH), total testosterone (T), estradiol (E2), sex hormone-binding globulin (SHBG), free androgen index (FAI = T/SHBG. 100), T/LH and T/E2 ratios, prolactin (PRO), parathyroid hormone (PTH), osteocalcin (OCN) between and within two groups pre and post intervention in asthenospermia infertile men.
3. Changes in the mean serum and seminal levels of calcium and serum phosphorus between and within two groups, pre and post intervention in asthenospermia infertile men.

### Supplements

The vitamin D3 supplements and placebo (containing maltodextrin) will be supplied by the “Pharmaceutical Technology Development Center of Ahvaz Jundishapur University of Medical Sciences, Iran”.

### Ethical considerations and trial registration

The protocol of this study was approved by the “Medical Ethics Committee of Ahvaz Jundishapur University of Medical Sciences” (approval no.: IR.AJUMS.REC.1396.1013) considering its accordance with the Declaration of Helsinki and was also registered at “Clinical Trials Registry of Iran” (IRCT20151128025274N4). A written consent will be obtained from all patients before starting the study. All patients’ data will be held confidential. This study will be financially supported by the Vice-Chancellor for Research Affairs of Jundishapur University of Medical Sciences, Ahvaz, Iran.

### Sample size

The sample size was calculated based on the progressive sperm ratio as the primary variable [29]. Considering a type I error of 0.05 and 90% for the power study, 34 patients were determined for each group. Also, regarding with the attrition rate of 25%, 43 patients will be included for each group. The formula used for the sample size was as follow:

$$ n=\frac{{\left({z}_1-\frac{\alpha }{2}+{z}_1-\beta \right)}^2\left({\delta_1}^2+{\delta_2}^2\right)}{{\left({\mu}_1-{\mu}_2\right)}^2} $$

### Randomization and blinding

The study randomization procedure will be done by a third person who will not be involved in this study. Each patient will receive a randomization code (determined through a computer-generated schedule). The random permuted blocks (15 blocks of 6) will be used to develop the randomization table. Vitamin D3 or placebo containers will be encoded based on the random codes. The odd and even numbers will be randomly allocated to groups A or B. The containers of vitamin D3 and placebo will be matched in appearance. As the containers will only be labeled with the codes without any further information, the patients will be blind in terms of intervention. The codes related to each group will be opened at the end of the study. In the present study both participants, the researchers and also statistical counselor will be blind in terms of the intervention.

### Withdrawal of participants

Participants who may withdraw prior to the randomization (during baseline phases I and II), will be replaced with new eligible participants. The exclusion criteria in this study would be as: have any acute illness, use less than 90% of the prescript supplement, would like to leave the study for personal reasons, participate in another study, immigrate and not available for following-up.

### Recruitment process

The study subjects will be selected from men referred to the infertility clinic of Ahvaz Jahad daneshgahi. After reading and reviewing the medical records of men referred to the center, the men with the primary inclusion criteria will be contacted by phone for an initial screening. The individuals interested in participating in the study will be invited for an in-person visit for further assessments.

### Procedures

**Fig. 2:**
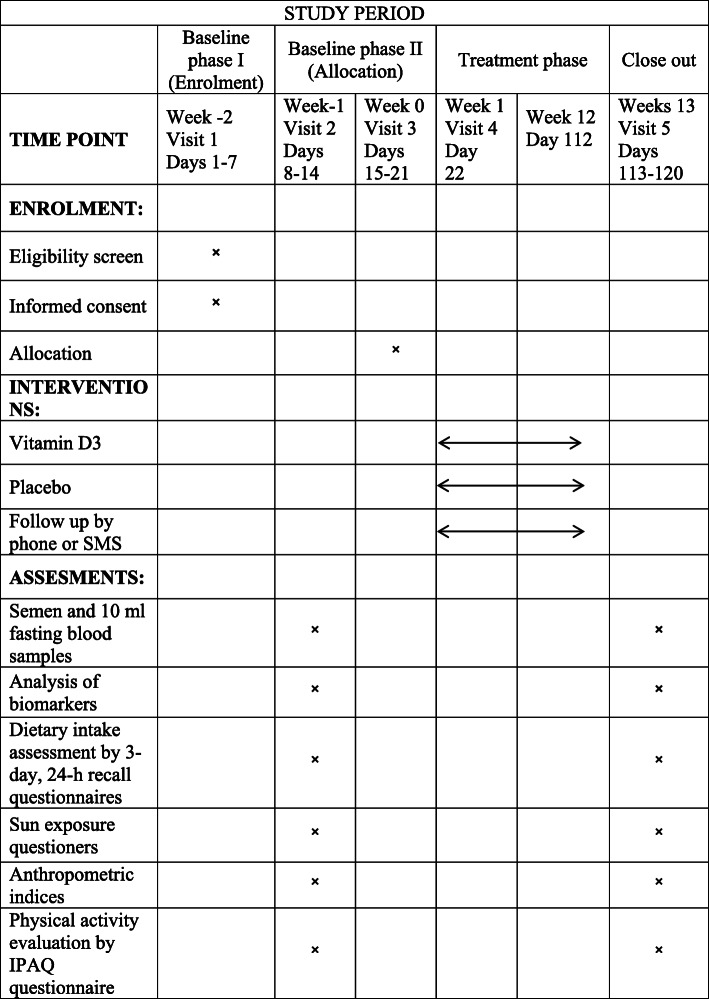
The schedule for enrollment, intervention and assessment based on the Standard Protocol Items; Recommendations for Interventional Trials (SPIRIT) Figure

The study flow diagram is given in Fig. 1. See additional file 1 for the Standard Protocol Items: Recommendations for Interventional Trials (SPIRIT) checklist. Questionnaires, measurements, and procedures as outlined in Fig. 2 (the SPIRIT diagram). The “Infertility Clinic of Ahvaz Jahad Daneshgahi Laboratory” will perform all laboratory analysis including analysis of blood and semen samples. The study visiting procedures are described in the following subsections.

### Baseline Phase I (Enrolment Phase): − 2 week

During visit 1, the final eligibility will be determined and a written consent will be obtained from participants by trained study staff. The information such as the procedures of the study, risks, side effects, confidentiality, voluntary participation, and right to withdraw will be provided to all participants. After consent is obtained, the study staff screens for drug abuse, obtains medical and concomitant medication histories, with eligibility determination based on the inclusion/exclusion criteria listed in Table 1. The necessary training to complete 3-day, 24-h recall questionnaires (including two week days and one weekend day) are given by a nutritionist to the participants and are asked to deliver them to the researcher during the second visit. In addition, participants are asked 2 to 7 days sexual abstinence to take semen samples. Semen and blood samples will be taken at the second visit.

### Baseline Phase II (Allocation Phase): 2 weeks (− 1, 0 weeks)

Once a participant is determined eligible, further demographic data, dietary intake, physical activity, sun exposure, anthropometric indices will be assessed, and blood and semen samples will be obtained during visit 2.

Demographic characteristics of patients are included: age (y), marriage duration (y), infertility duration (y), education, race, physical activity, sun exposure, time of sun exposure, sun exposure area, and sun screen use. The dietary intake will be evaluated using a 3-day, 24-h recall questionnaires (including two week days and one weekend day). Total energy, macronutrient, and micronutrient intake will be estimated using nutritionist IV software. The short form of the international physical activity questionnaire (IPAQ) will be used to evaluate physical activity levels [53]. The sun exposure time will be assessed with a questionnaire [54]. Anthropometric indices will be measured after overnight fasting, with minimal clothing and without shoes. Body weight and body fat percent will be measured with light clothes, no shoes and accuracy of 0.1-kg by Omron scale (Germany). Height will be measured without shoes with the accuracy of 0.5-cm by a stadiometer. Waist and hip circumferences will be measured by measuring meters (Seca, Germany). BMI will be computed by dividing the weight (kg) by the height squared (m^2^).

### Semen and blood samples

Semen samples will be obtained after 3 days of sexual abstinence. All semen samples will be held at 37C until the liquefaction. The sperm quality parameters such as ejaculate volume, total sperm count, sperm concentration, motility grade and sperm with normal morphology (%) will be assessed according to the WHO criteria [55]. The other biochemical analysis such as seminal levels of oxidative stress markers including malondialdehyde (MDA), 8-hydroxy-2- Dioxy Guanosine (8-OHDG) and total antioxidant capacity (TAC) and calcium will also be analyzed. Sperm DNA fragmentation will be assessed using sperm chromatin dispersion (SCD) method [11, 12].

Moreover, 10 cc of intravenous blood samples will be collected from patients to analyze biochemical parameters including 25 hydroxyvitamin D (25-OHD), oxidative stress index (TAC and MDA) and 8-OHDG, Sex hormones (total testosterone, estradiol (E2), luteinizing hormone (LH), follicle-stimulating hormone (FSH), sex hormone-binding globulin (SHBG), prolactin), parathyroid hormone (PTH), osteocalcin, calcium, and phosphorus.

The measurement of serum calcium and phosphorus and seminal calcium will be done using calorimetric method. Seminal and serum levels of vitamin D, TAC, MDA, 8-OHDG, PTH, osteocalcin, Testosterone, E2, SHBG, luteinizing hormone (LH), follicle-stimulating hormone (FSH), and prolactin will be assessed by enzyme-linked immunosorbent assay (ELISA).

During Baseline Phase II, the participants will be excluded if they have:

1. Body mass index (BMI) > 30 kg/m^2^
2. Normal semen samples analysis for sperm motility (the mobility of sperm > 40% and rapid progressive sperm motility> 32%)
3. Sufficient serum levels of 25OHD levels (> 30 ng / ml)
4. Intense physical activity (> 3000 met/cal/week)

In visit 3 patients will be randomly allocated to either group A, receiving 4000 IU/d [51, 52] vitamin D3, or group B, receiving a matched placebo for 12 weeks (Randomization and blinding method described above).

### Treatment phase (12 weeks)

In this phase (visit 4), the patients will be asked to use vitamin D3 and placebo supplements one pill every day for 12 weeks. Also, the patients will be asked not to change their diet, not to participate in any other clinical trials, and not to take any medications or supplements without consulting the researchers during this time. The compliance of patients to use the supplements will be done through either contacting by phone or sending a phone message every day in this phase.

### Follow up phase (12 weeks after baseline)

In visit 5, all measurements such as anthropometric indices, physical activity, dietary intake, and sun exposure will be assessed and blood and semen samples will be collected.

### Compensation

All supplements and tests will be free of charge for participants in this study. Also, the transportation costs will be paid. In addition, at the end of the study, free nutritional counseling will be provided to all patients.

### Data management and monitoring

Participants’ information, all forms completed by them and their test results will be held confidential throughout all stages of the research cycle and biological samples will be stored in a protected area with limited access. Trial data will be checked in regard of accuracy and following standard operating procedures and policies during study, by an independent data management team at Ahvaz Jundishapur University of Medical Sciences.

### Statistical analysis

Intention-to-treat (ITT) and per-protocol (PP) populations will be used in the analysis. The ITT population consists of all individuals who will be randomized, whereas the PP population consists of all participants who complete the 12-week intervention. All data will be analyzed using SPSS (Version 22; SPSS Inc., Chicago, IL). Results will be expressed as mean ± SD and frequency and (percentage) for quantitative and qualitative variables respectively. The normal distribution of variables will be tested and confirmed by Kolmogorov-Smirnov test. The baseline differences of mean values between groups will be tested using independent sample t-test or Mann-Whitney U test for normal and non- normal distribution variables respectively. Analysis of covariance (ANCOVA) will be used to identify any differences between the two groups at the end of study, adjusting for baseline values and covariates (such as BMI, age, dietary intake including dietary calorie, calcium, F, selenium, vitamin C, E, A, D and zinc, physical activity, sun exposure, season, serum vitamin d, PTH and calcium). The comparison of mean values within groups pre- and post-intervention will be done by paired sample t tests and Wilcoxon signed-rank test as nonparametric alternatives for normal and non- normal distribution variables respectively. Differences will be considered statistically significant at *P* < 0.05.

### Safety, adverse effects and monitoring data

According to IOM studies the dosage of 4000 IU/day is in the safe range for vitamin D [51, 52] and no side effects reported. However, any possible side effects will be reported to the Ethics Committee of the Ahvaz University of Medical Sciences.

## Discussion

The expression of vitamin D receptors and metabolizing enzymes in the testis and spermatozoa indicate the key role of vitamin D in male reproductive system [22]. However, there is still no general consensus on the role of vitamin D in male fertility.

Some evidence suggests that OS plays an independent role in the etiology of male infertility, with 30 to 80% of infertile men having elevated seminal ROS levels [56]. The increased ROS along with decreased antioxidant defense can result in redox imbalance, reduced sperm motility and sperm DNA damage [57]. In animals with Vitamin D deficient diet and diet-induced obesity, there was significant increase reported in DFI of spermatozoa [28]. However, the only human observational study [58] did not find such a relationship.

The antioxidant [59, 60] and anti-inflammatory [61–63] role of Vitamin D has been previously shown in various studies. Vitamin D may improve OS and protect macromolecules such as DNA and cell membranes against the oxidative damage and may improve sperm motility. The association between Vitamin D deficiency and low semen quality has been reported in several animal and human studies [25–27, 64–66]. Also, calcium mediated effects of vitamin D on increasing motility and upward migration of spermatozoa have been reported in several animals and experimental studies [27, 64, 66–70].

There are only few interventional studies evaluated the effects of vitamin D on sperm parameters with controversial findings. Deng XL et al. study showed that 3 months supplementation with 200 IU/d vitamin D and 600 mg calcium in oligo-asthenozoospermia infertile men significantly increased the sperm progressive motility and rate of pregnancy in the intervention group [29]. In contrast, in the other randomized clinical trials carried out by Blomberg Jensen et al. [30] and Amini et al. [31] there were no significant differences observed in sperm parameters between two groups post intervention.

In addition, the effects of vitamin D deficiency on serum levels of testicular hormones have been assessed by several animals and observational studies with controversial results [32–39]. There was no clear relationship found between vitamin D status and production of testicular hormone according to these studies. However, it was proposed that there may be an indirect effect of vitamin D on T synthesis, mediated by a genomic vitamin D induced expression of osteocalcin (a hormone synthesized by osteoblasts and involved in the metabolism of bone) [45, 71–73], and SHBG [32, 45, 46, 74, 75] and a direct effect of vitamin D on T secretion probably through the genomic vitamin D induced expression of calbindinD28K (a calcium-binding protein regulates calcium homeostasis [76]) and calcium and phosphate homeostasis [75].

The results of available interventional studies regarding with the effects of vitamin D on serum levels of T are controversial. There are some studies showed no positive effects of vitamin D supplementation on serum T levels. A study with very short-duration (4 days) and a study with short-duration (12 weeks) of vitamin D supplementations found no significant effects on serum total T levels [40, 41]. Whereas, in the other studies with a long duration of vitamin D supplementation (12 months), there were significant increased serum total levels of Tin men with different age [39], free T and SHBG [42]. Moreover, in the other studies with 12 and 24 months supplementation [43], there were no effects seen. It is suggested that the duration of intervention, the dosage of vitamin D supplement and different characteristics of the subjects such as age, BMI, having diseases, serum levels of 25-OHD and the presence of other confounding factors including the time of blood sampling, serum levels of LH, SHBG and calcium may cause the controversial results.

There are also controversial results for the impact of vitamin D on serum levels of estradiol. No significant relationship was observed between serum levels of E2 and 25-OHD in some cross-sectional studies carried out in healthy young men [33, 45, 46]. On the other hand, negative correlation was seen between serum E2 and 25-OHD and ionized calcium in several studies [47–49]. It has been recently shown that the increased T/E2 ratio is more important for male fertility than T or E2 levels alone [77]. In Andersson et al. study, a low T/E ratio was observed in a population with infertile men [78].

Furthermore, a higher level of prolactin along with low levels of vitamin D has been demonstrated in infertile men. The increased prolactin can inhibit spermatogenesis, damage sperm motility and reduce sperm quality through inhibition of the pulsating cycle of gonadotropin and T secretion [79, 80].

We believe that the strengths of this study are as follow: the daily dosage of vitamin D, the wide range of exclusion criteria such as smoking and having any specific disease, control of confounding factors including BW, BMI, BF%, WC, physical activity, season, dietary calorie, calcium, F, VD3, selenium, vitamin C, E, A, zinc, serum 25-OHD and PTH and considering baseline values. However, there are some limitations in this study including not selecting healthy subjects, not measuring ROS, endogenous antioxidants such as glutathione, selenium, and vitamin E and some other hormones such as inhibin. In addition, one of the possible operational issues in this study is a high probable rate of participants withdraw. For this reason a high dropout percentage (25%) will be considered in terms of calculating the sample size.

Therefore, due to the role of OS in the pathogenesis of idiopathic male infertility and also considering the antioxidant property of vitamin D and probable positive relationship between semen quality and vitamin D levels in men and since there is no clinical study on the effect of vitamin D supplementation on OS markers in men with asthenospermia and insufficient vitamin D levels or vitamin D deficiency, and also regarding with the high prevalence of vitamin D deficiency in infertile men, it seems that further clinical studies are needed in this area before any recommendation to use vitamin D supplement as part of the treatment of male infertility. Therefore, this RCT will be done to investigate the effects of the vitamin D supplementation on sperm parameters, seminal and serum levels of oxidative stress and serum endocrine factors in asthenospermia infertile men.

### Trial time-scale

The protocol was approved in 2018 and the recruitment was started in April 2018. The trial is in the enrolment stage in 2021. It is predicted that the patient recruitment will be completed in September 2021.

## Supplementary Information

**Additional file 1.** SPIRIT 2013 Checklist: Recommended items to address in a clinical trial protocol and related documents*

## Data Availability

The datasets of this study will be available at the end of the study and the results will be published and provided by the corresponding author following a formal request.

## Abbreviations

ANCOVA: Analysis of covariance
BMI: Body mass index
DNA: Deoxyribonucleic acid
DFI: DNA fragmentation index
E2: Estradiol
ELISA: Enzyme-linked immunosorbent assay
8-OHDG: 8-hydroxydeoxyguanosine
FSH: Follicle-stimulating hormone
IPAQ: International physical activity questionnaire
LH: Luteinizing hormone
MDA: Malondialdehyde
OS: Oxidative stress
PTH: Parathyroid hormone
ROS: Reactive oxygen species
RCT: Randomized controlled clinical trial
SCD: Sperm Chromatin Dispersion
SHBG: Sex hormone-binding globulin
TAC: Total Antioxidant Capacity
WHO: World Health Organization
